# Education, Employment, Income, and Urban–Rural Differences as Drivers of Social Inequalities in Environmental Exposures: Evidence from the UK Biobank

**DOI:** 10.1021/acs.est.5c08849

**Published:** 2026-01-22

**Authors:** Gauthier Pereira, Benoît Lepage, Kees de Hoogh, Ruben Colindres Zuehlke, Fernando Guntoro, Lola Neufcourt, David Tang, Rin Wada, Roel Vermeulen, Michelle Kelly-Irving, Cyrille Delpierre, Marc Chadeau-Hyam, Raphaële Castagné

**Affiliations:** † EQUITY Team, Centre d’Epidémiologie et de Recherche en santé des POPulations CERPOPUMR1295, Inserm−Université de Toulouse, 31400 Toulouse, France; ‡ 30247 Swiss Tropical and Public Health Institute, 4123 Allschwil, Switzerland; § University of Basel, 4001 Basel, Switzerland; ∥ Department of Epidemiology and Biostatistics, School of Public Health, 4615 Imperial College London, London W12 0BZ, U.K.; ⊥ Institute for Risk Assessment Sciences, Utrecht University, 3584 CM Utrecht, The Netherlands

**Keywords:** external exposome, socioeconomic position, social and life course epidemiology, environmental exposures, cohort study, socially patterned exposures

## Abstract

This study investigates the social patterning of environmental exposures, examining how three socioeconomic position dimensions influence exposure to air pollution, road traffic noise, and availability of green spaces, in both urban and rural areas in England, Scotland, and Wales. Using data from the UK Biobank cohort study, we assessed associations between three individual markers of socioeconomic position: educational attainment, household income, and employment status, and three environmental exposure domains: residential airborne pollutants, residential road traffic noise, and residential green and blue space. In urban areas, participants with lower educational attainment, lower household income, or those who were unemployed exhibit a higher exposure to airborne pollutants. Household income also influences the vicinity to green spaces and natural environments, with lower incomes experiencing less green and natural environments in both the rural and urban contexts. Retired individuals experienced lower exposure to airborne pollutants and lived in areas with more green spaces or natural environments compared with employed individuals. These patterns were consistent across England, Scotland, and Wales, although some geographical and national differences were observed. The study highlights the complex interplay among socioeconomic factors, geographical location, and environmental exposures. Our findings suggest that socioeconomic position is a key determinant of specific external exposomes, with socioeconomically disadvantaged groups experiencing more adverse environmental conditions.

## Graphical abstract

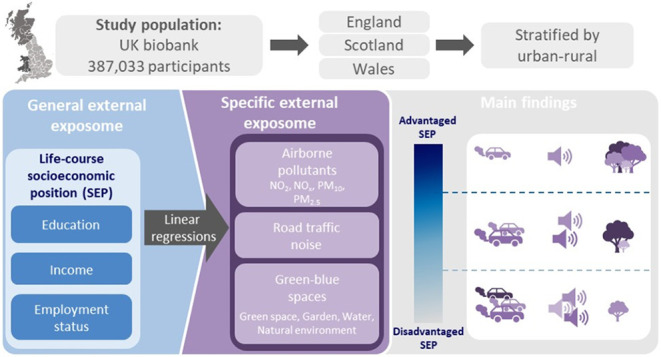

## Introduction

Social inequalities in health refer to the phenomenon whereby individuals in disadvantaged socioeconomic circumstances are more likely to suffer from ill health and premature death.^1^ Health inequalities^2^ are not entirely explained by behavioral risk factors^3^ and access to healthcare,^4^ suggesting that other pathways are involved. Specifically, the social determinants of health, defined as “the conditions in which people are born, grow, live, work, and age, and the wider set of forces and systems shaping the conditions of daily life” World Health Organization (WHO),^5^ play a significant role in shaping a person’s lived experience. This definition aligns closely with the definition of the exposome, as originally introduced by Wild in 2005.^6^ We previously reported that although the social environment plays a crucial role in shaping the general external exposome, it remains insufficiently explored compared to the specific external exposome (chemical agents, environmental pollutants, noise and lifestyle factors) and the internal exposome (-omics, metabolic processes, circulating blood biomarkers).^7^

In parallel, ongoing research attempts to unravel how social exposures are embodied, ultimately shaping health and contributing to the construction of social inequalities in health over the life course.^8,9^ Building on works by Krieger,^10–12^ Hertzman,^13,14^ Elder,^15^ and Blane et al.,^8,9^ Kelly-Irving and Delpierre^16^ proposed a framework to understand social inequalities in health through social-to-biological mechanisms. This framework explains how social factors cause biological changes, identifying two main types of socially distributed mechanisms: (1) mechanism of “exogenous origin” where external factors like pollutants, food, or physical activity trigger biological responses; and (2) mechanism of “endogenous origin” where interactions with the environment trigger internal biological responses, mainly linked to stress perception and stress response systems.^16^ The social-to-biological transition framework bridges the three domains of the exposome (general external exposures, specific external exposures, and internal exposures), leading to the conception of the socio-exposome framework that also incorporates the dimension of environmental justice.^17^

A global review of environmental inequality by socioeconomic position in relation to airborne pollutants found that, overall, socioeconomically disadvantaged individuals are exposed to higher levels of air pollution in studies from North America, while European findings are more heterogeneous.^18^ Similarly, Dreger et al., focusing on the WHO European Region, reported that in Europe socioeconomically disadvantaged groups are disproportionately exposed to environmental noise, although findings vary across different regions and contexts.^19^ Schüle et al. reported that socioeconomically deprived areas have lower availability of green space compared to more affluent areas, whereas findings from individual-level analyses reveal more heterogeneous associations across specific socioeconomic indicators.^20^

Growing evidence also highlights the adverse effects of exposure to poorer environments on health. Exposure to airborne pollutants, such as particulate matter (PM_2.5_) and nitrogen oxides have been found to be associated with respiratory and cardiovascular diseases.^21^ Chronic exposure to ambient noise—from road, railway, or aircraft noise—have also been found to be associated with poorer mental health^22^ and increased risk of hypertension.^23^ In contrast, the availability of green spaces has been shown to have a protective effect against depression^24^ and mortality.^25^ Taken together, these findings suggest that environmental exposures play a pivotal role in shaping social inequalities in health.

Most studies of social inequalities in environmental exposures have employed ecological designs based on area-level deprivation indices. Individual-level studies have often focused on single socioeconomic indicators and have rarely examined multiple environmental exposures. However, urban exposome studies have examined both area-level and individual-level socioeconomic indicators, particularly in studies involving pregnant women^26^ and children.^27^ To address these gaps, we use data from the UK Biobank study to examine the social patterning of environmental exposures across urban and rural areas in England, Scotland, and Wales. Our analysis focuses on three socioeconomic markers at the individual level at different life stages, educational attainment, employment status, and household income, and their associations with three major domains of environmental exposures, air pollution, noise traffic pollution, and availability of green space and blue spaces. We hypothesize that individuals with disadvantaged SEP are more likely to experience more adverse environmental conditions and that the social patterning of environmental exposures may differ between urban and rural contexts.

## Materials and Methods

### Study Design

The UK Biobank is a large-scale prospective study of 502,366 individuals aged 37–73 years at recruitment (between 2006 and 2010) from 22 assessment centers in England (17), Scotland (2), and Wales (3). Information on socioeconomic characteristics, lifestyle, and health was collected by a self-administered touch-screen questionnaire and face-to-face interview.^28^ UK Biobank was established in accordance with the Declaration of Helsinki. All participants in the UK Biobank provided informed consent. This study includes only those who maintained their consent throughout, excluding 425 individuals who withdrew it. The UK Biobank Study received approval from the National Information Governance Board for Health and Social Care and the National Health Service North West Multicenter Research Ethics Committee. Our study was conducted under application number 69328.

### Inclusion/Exclusion Criteria

We excluded participants with incomplete information on the country of residence, socioeconomic characteristics, environmental exposures, and urbanicity, leaving a total of 387,033 UK Biobank participants in the final analyses (Supporting Figure S1).

### Theoretical Framework of Our Study

A conceptual model was constructed based on existing literature on directed acyclic graphs (DAG)^29,30^ to guide our statistical analyses and define our models. This conceptual framework, presented in Figure 1, represents our literature-based assumptions regarding the social patterning of outdoor environmental exposure and its underlying mechanisms. Environmental exposure may be a pathway through which the social environment negatively affects health and contributes to social inequalities in health. Urban and rural areas exhibit substantial differences in environmental exposures: in urban areas, exposures to air pollutants are typically higher due to factors such as industrial activities, vehicular emissions, and greater population density. Similar patterns are observed for road traffic noise, whereas rural areas generally provide greater access to natural green spaces but may be subject to other sources of environmental exposure, such as agricultural activities, biomass burning, and industrial activities located outside urban areas. Additionally, we conceptualized urbanicity as a structural determinant of socioeconomic position within our analytical framework. This assumption reflects the significant differences in socioeconomic structures, opportunities, and available resources between urban and rural areas. Long-term evidence from England indicates that urban areas have consistently higher levels of deprivation than rural areas over the period 1971 and 2020.^31^ These contextual disparities are also observable in our sample: individuals living in urban areas tend to have lower educational attainment, are more likely to be employed, and report lower household income compared with those in rural settings (Table 1). For these reasons, we treat urbanicity as an upstream determinant influencing both socioeconomic position and environmental exposures. Based on these differences, our first methodological choice was to stratify analyses in urban and rural settings. We next focused on three socioeconomic position (SEP) indicators at the individual level: education, employment status, and income, as they capture two distinct life stages and different dimensions of SEP.^32,33^ Our study examined outdoor environmental exposures, including air pollution, noise, and access to green and blue spaces.

**Table 1:** Descriptive Statistics of Participants Characteristics by Countries and Living Area in Our UK Biobank Analytical Sample (*N* = 387,033)

		England			Scotland			Wales		
		urban	rural	*p*	urban	rural	*p*	urban	rural	*p*
variables		*n* = 292,246	*n* = 50,178		*n* = 25,591	*n* = 2401		*n* = 14,089	*n* = 2528	
sex	female, *n* (%)			0.336^a^			0.498^a^			0.174^a^
		154,411 (52.84)	26,395 (52.6)		13,918 (54.39)	1288 (53.64)		7377 (52.36)	1286 (50.87)	
	male, *n* (%)	137,835 (47.16)	23,783 (47.4)		11,673 (45.61)	1113 (46.36)		6712 (47.64)	1242 (49.13)	
age				<.001^b^			<.001^b^			<.001^b^
	median (IQR)	57 (50-63)	59 (51-63)		57 (49-63)	58 (50-64)		57 (49-62)	58 (51-63)	
country of birth				<.001^a^			<.001^a^			<.001^a^
	UK or Ireland, *n* (%)	268,161 (91.76)	48,172 (96)		24,499 (95.73)	2325 (96.83)		13,496 (95.79)	2448 (96.84)	
	elsewhere, *n* (%)	24,085 (8.24)	2006 (4)		1092 (4.27)	76 (3.17)		593 (4.21)	80 (3.16)	
educational attainment				<.001^a^			<.001^a^			<.001^a^
	high, *n* (%)	100124 (34.26)	19559 (38.98)		10960 (42.83)	872 (36.32)		4706 (33.4)	965 (38.17)	
	medium, *n* (%)	98,956 (33.86)	17,416 (34.71)		7613 (29.75)	827 (34.44)		4792 (34.01)	879 (34.77)	
	low, *n* (%)	93,166 (31.88)	13,203 (26.31)		7018 (27.42)	702 (29.24)		4591 (32.59)	684 (27.06)	
household income				<.001^a^			<.001^a^			<.001^a^
	greater than 52,000, *n* (%)	73,662 (25.21)	16,558 (33)		7499 (29.3)	607 (25.28)		3234 (22.95)	729 (28.84)	
	31,000 to 51,999, *n* (%)	77,290 (26.45)	13,893 (27.69)		7090 (27.71)	748 (31.15)		3989 (28.31)	746 (29.51)	
	18,000 to 30,999, *n* (%)	76,275 (26.1)	12,290 (24.49)		6190 (24.19)	600 (24.99)		3818 (27.1)	674 (26.66)	
	less than 18,000, *n* (%)	65019 (22.25)	7437 (14.82)		4812 (18.8)	446 (18.58)		3048 (21.63)	379 (14.99)	
employment Status				<.001^a^			<.001^a^			<.001^a^
	employed or self-employed, *n* (%)	173,423 (59.34)	27,496 (54.8)		15566 (60.83)	1398 (58.23)		8635 (61.29)	1430 (56.57)	
	retired, *n* (%)	100,510 (34.39)	19,612 (39.08)		8618 (33.68)	880 (36.65)		4702 (33.37)	950 (37.58)	
	unemployed, *n* (%)	18,313 (6.27)	3070 (6.12)		1407 (5.5)	123 (5.12)		752 (5.34)	148 (5.85)	
NO_2_				<.001^b^			<.001^b^			<.001^b^
	median (IQR)	27.4 (23.3-32)	16.6 (14.4-19.1)		27.9 (24.2-31.8)	17.9 (15.6-20.5)		26 (22.5-29.7)	17.7 (14.9-20.4)	
NO_*x*_				<.001^b^			<.001^b^			<.001^b^
	median (IQR)	43.9 (37-52)	26.7 (22.3-33.5)		45.4 (38.8-51.6)	30 (24.4-36.4)		42.9 (36.3-50.1)	28.9 (23.2-36)	
PM_10_				<.001^b^	NOT AVAILABLE					<.001^b^
	median (IQR)	16.1 (15.5-17)	14.8 (13.2-16)					16.1 (15.6-17.3)	15.2 (13.7-17.7)	
PM_2.5_				<.001^b^						<.001^b^
	median (IQR)	10.1 (9.5-10.7)	8.7 (8.2-9.2)					10.1 (9.5-10.7)	8.8 (8.3-9.4)	
lday				<.001^b^			<.001^b^			<.001^b^
	median (IQR)	54.2 (52.9-56.1)	54.5 (52.6-57.2)		54.4 (53.2-56.1)	54.3 (52.4-56.6)		54.4 (53-56.4)	54.7 (52.7-57.9)	
levening				<.001^b^			<.001^b^			<.001^b^
	median (IQR)	50.5 (49.1-52.4)	50.7 (48.8-53.4)		50.6 (49.5-52.4)	50.5 (48.7-52.9)		50.6 (49.2-52.6)	50.9 (48.9-54.1)	
lnight				<.001^b^			<.001^b^			<.001^b^
	median (IQR)	45.4 (44-47.3)	45.6 (43.8-48.4)		45.6 (44.4-47.3)	45.5 (43.6-47.8)		45.6 (44.2-47.5)	45.9 (43.8-49.1)	
natural environment				<.001^b^			<.001^b^			<.001^b^
	median (IQR)	32.1 (17.6-49.9)	84.2 (73-93)		26.2 (15.5-44.8)	78.2 (64.6-87.1)		34 (18.2-51.8)	81.9 (69.8-90.7)	
greenspace				<.001^b^	NOT AVAILABLE			NOT AVAILABLE		
	median (IQR)	37.5 (25.9-52.1)	81.4 (71.6-89.6)							
garden				<.001^b^	NOT AVAILABLE			NOT AVAILABLE		
	median (IQR)	26.5 (19.9-33.5)	8.6 (4.3-14.2)							
water				<.001^b^	NOT AVAILABLE			NOT AVAILABLE		
	median (IQR)	0.5 (0.2-1.2)	0.6 (0.3-1.4)							

a Chi-squared test.

b Wilcoxon rank test.

**Figure 1:**
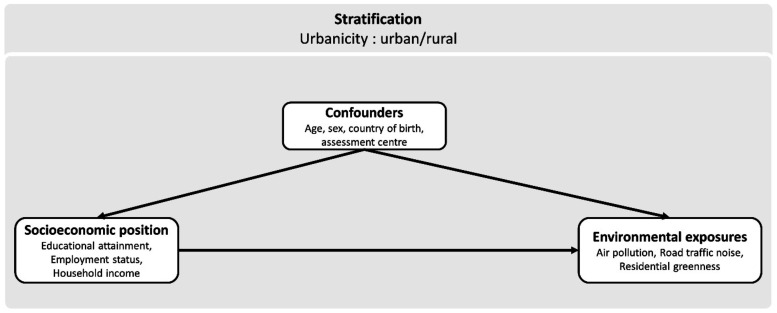
Conceptual model of the association between socioeconomic position and environmental exposures showing the included covariates.

### Socioeconomic Position

We used three individual-level socioeconomic position markers collected at baseline to cover different dimensions of socioeconomic position at two life stages: in young adulthood and in middle-to-late adulthood. Young adulthood SEP was measured through each participant’s own educational attainment, which was recoded into three categories based on the International Standard Classification of Education level^34^ as “high” [College or University degree], “medium” [NVQ or NHD, HNC or equivalent, A levels/AS levels or equivalent, Other professional qualifications eg: nursing, teaching], and “low” [O levels/GCSEs or equivalent, CSEs or equivalent, None of the above]. Middle-to-late adulthood SEP was proxied by participant’s employment status as “employed” [paid employment or self-employed], “retired” [retired], and “unemployed” [looking after home and/or family, unable to work, unemployed, unpaid, or voluntary work or student], and annual household income (GBP/year) coded in four categories as <18,000; 18,000–30,999; 31,000–51,999 and >52,000 (Supporting Table S1).

### Environmental Exposures

Previous studies have modeled environmental exposures and joined them with the UK Biobank. Participants’ home addresses at recruitment were geocoded and linked to estimations of residential environmental exposures. We considered three domains of environmental exposures: residential airborne pollutants, residential road traffic noise, and residential green and blue space.

#### Residential Airborne Pollutants

Residential air pollutants were estimated using Land Use Regression (LUR) developed as part of the ESCAPE project for the year 2010.^35,36^ Briefly, the LUR model uses geographic information systems (GIS)-derived predictor variables such as traffic intensity and distance to the road to approximate the annual air pollutant concentration at the home address. We examined annual average concentrations (μg/m^3^) of nitrogen dioxide (NO_2_), oxide of nitrogen (NO*_X_*), as well as the concentration of particle matter with a diameter <2.5 μm (PM_2.5_) and <10 μm (PM_10_).

#### Residential Road Traffic Noise

The CNOSSOS-EU (Common NOise aSSessment MethOdS) noise model was used to estimate the residential road traffic noise in 500 m radius from participants’ residential addresses for the year 2009.^37,38^ The CNOSSOS model accounts for factors such as noise propagation, absorption, and distance to the roads, as well as traffic flow or type of vehicles, to estimate the residential annual average sound level. We investigated the annual average sound level in decibels (dB­(A)) of road traffic noise during daytime (average sound level from 07:00 to 19:00), evening (average sound level from 19:00 to 23:00), and nighttime (average sound level from 23:00 to 07:00).

#### Residential Green Space and Blue Space

Generalized land use database (GLUD 2005), which provides a measure of residential green space for England in 2005, was combined with the 2001 census output areas (COA) obtained from the neighborhood statistics to full spatial resolution. Output areas are the lowest geographical unit and have approximately 100 to 625 resident individuals. For each COA, the percentage of land categorized as green space (green space%), domestic garden (garden%), and water (water%) was calculated. To assign these land uses to individuals, a 1000 m buffer was created around each participant’s residential address. The buffer was then intersected with the COA in order to calculate an area-weighted mean of each land use for each participant. Types of land classified as green spaces include parks, agricultural land, and other open space measures both includes. As GLUD measures were only available in England and in order to have one harmonized measure of green space for the three countries, the land coverage estimate for the natural environment (natural environment%) was also included. Natural environment was obtained from the 2007 Raster Land Cover Map (LCM) for 25m grid cells in England, Scotland, and Wales in 2007.^39^ LCM provides 23 classes that were recategorized into a binary classification: natural environment (classes 1–21) and built environment (classes 22–23). Natural environment can be considered equal to green space as it includes notably grassland, woodland, agricultural land, and littoral spaces, and excludes built-up areas and gardens.

Supporting Table S2 provides a summary of the environmental variables used in the analysis.

### Covariates

Age at recruitment (continuous), sex (“women”/”men”), country of birth (“UK or Ireland”/ “Elsewhere”), and center (22 modalities) were identified as confounders potentially affecting both the SEP and the exposure to environmental factors. Supporting Table S3 provides a summary of the covariates used in the analysis.

### Urban–Rural Residential Classification

As levels of environmental factors broadly vary across geographical contexts, we have conducted stratified analysis in urban and rural areas. We used an urban–rural variable based on participants’ postcode at recruitment and the urban/rural classification from the 2001 UK census. The categories for England, Wales, and Scotland are detailed in Supporting Table S4. These were simplified into 2 broader categories, ‘urban’ and ‘rural’, for analyses within England, Wales, and Scotland.

### Data Analysis

#### Descriptive Analysis

We calculated means and frequencies for all continuous and categorical baseline characteristics by country (England, Scotland, Wales) and by urbanicity (urban/rural). χ^2^ test for categorical variables and the Wilcoxon rank test for continuous variables were used to assess differences across subpopulations.

#### Regression Analysis

Linear regression models were estimated to investigate the relationships between each SEP indicator and environmental exposure by urban–rural residential localization in England, Scotland, and Wales. We defined the most advantaged SEP as the reference category [“high educational attainment” for education, “being employed or self-employed” for employment status, and a “income > £52,000/year” for income].

Our model was correcting for age, sex, country of birth, and assessment center. Analyses were conducted on each SEP (3), environmental exposures (11), countries (3), and residential area localization (2) combinations, separately resulting in 198 models. Stringent correction for multiple testing was achieved using the Bonferroni approach, setting the per-test significance level to resulting in a threshold of *P* = 0.05/198 (*P* = 0.00025), and controlling the family-wise error rate <0.05.

#### Additional Analyses

To assess whether the association between household income and each environmental exposure in England, stratified by urbanicity (urban/rural), was explained by educational attainment, employment status, or both, we sequentially controlled for these two SEP indicators in a chronologically ordered manner, resulting in four models.

Model 1: Household income, adjusted for age, sex, country of birth, and center

Model 2A: Model 1 + educational attainment

Model 2B: Model 1 + employment status

Model 3: Model 1 + educational attainment + employment status.

All statistical analyses were performed using R version 4.1.3.

## Results

### Participant Characteristics

Our study included a total of 387,033 individuals (77.11% of the initial population) from three different countries. The proportion of individuals living in urban areas was relatively similar across countries: 85.3% in England, 84.8% in Wales, and up to 91.4% in Scotland (Table 1). In England and Wales, the population living in rural areas was observed to be more educated, have higher household income, and were more often retired compared to urban populations. Additionally, they were less exposed to airborne pollutants and had greater availability of green space and natural environment within a 1000 m radius of their residence’s address. Supporting S5 provides a description of participants’ characteristics in the analytical sample and in the full UK Biobank. Bivariate associations between environmental exposures and each SEP indicator, and covariates are presented in Supporting Tables S6 and S7 for participants living in urban and rural areas in England.

#### Association Between SEP Indicators and Environmental Exposures in Urban Environments in England

Results between each SEP indicator and environmental exposures in urban residential localization in England (*N* = 292,246) are presented in Figure 2 and Supporting Table S8.

**Figure 2:**
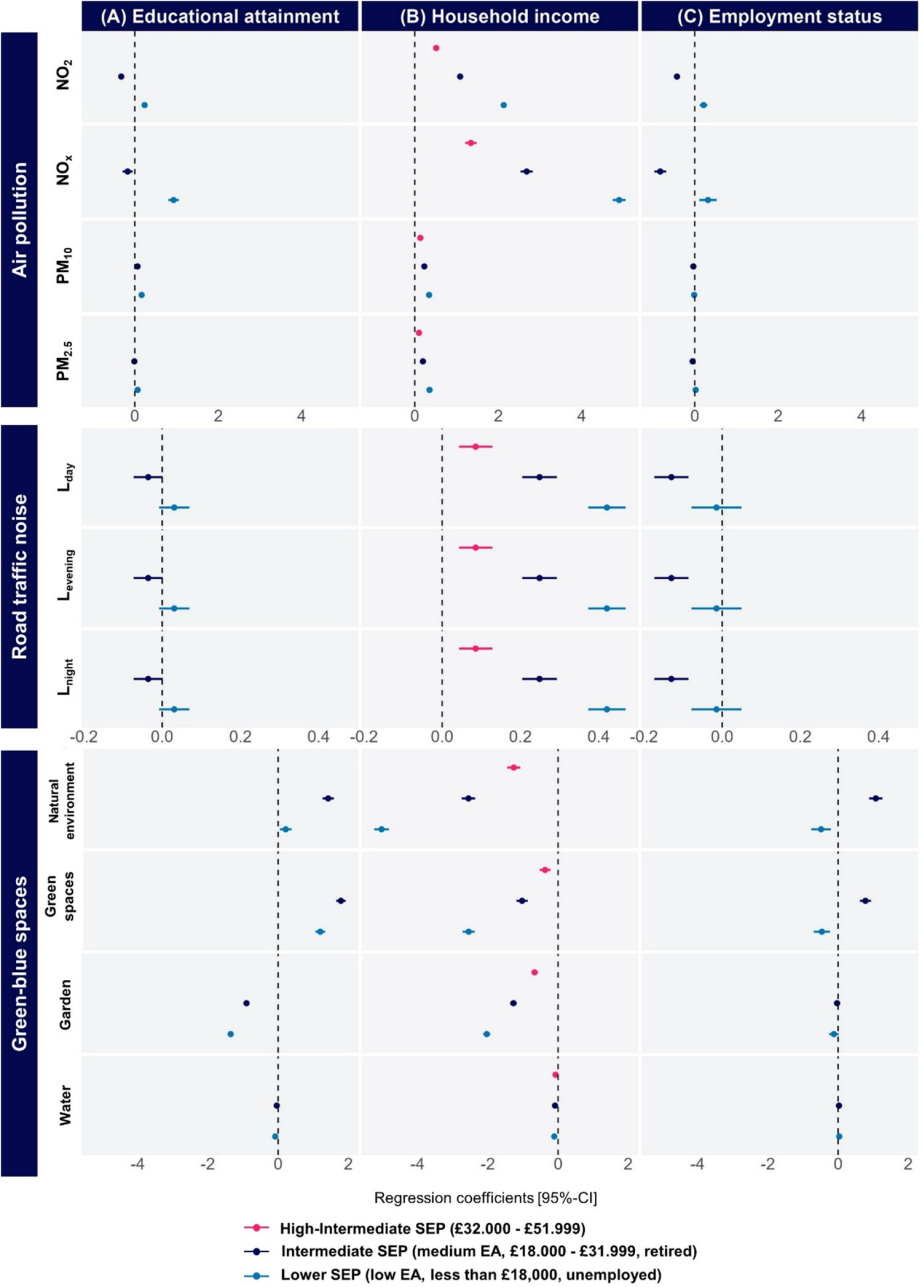
England urban areas: Forest plot of linear regression coefficients [95% confidence interval] for the association between (A) educational attainment, (B) household income, or (C) employment status and air pollution, road traffic noise, and green-blue spaces in urban areas for England (*N* = 292,246). β coefficients represent the difference in exposure to airborne pollutants, road traffic noise, and green-blue spaces compared to the reference group adjusted for covariates: for all environmental exposures, the most advantaged socioeconomic group was used as reference, and a positive association therefore indicates higher exposure in the less advantaged socioeconomic groups.

Individuals with lower educational attainment (EA) were found to have been exposed to higher levels of NO_2_, NO*_X_*, PM_10_ and PM_2.5_ compared to those with high EA (Low vs High EA: β_NO_2__ = 0.24 (95% CI 0.18; 0.29); β_NO*_X_*_ = 0.93 (95% CI 0.80; 1.05); β_PM10_ = 0.16 (95% CI 0.15; 0.18); β_PM2.5_ = 0.07 (95% CI 0.06; 0.08)). Associations between a medium level of education and exposures to airborne pollutants were generally weaker and in the opposite direction (Medium vs High EA: β_NO_2__ = −0.33 (95% CI −0.38; −0.27); β_NO*_X_*_ = −0.17 (95% CI −0.29; −0.05)). The relationship between educational attainment and road traffic noise was weak, with a small effect size observed (Figure 2A).

Additionally, individuals with a lower educational attainment were less likely to live in areas with a high percentage of gardens (Low vs High EA: β_garden,1000m_ = −1.35 (95% CI −1.43; −1.27)) and more likely to reside in areas with green spaces (Low vs High EA: β_greenspaces,1000m_ = 1.20 (95% CI 1.06; 1.34)). Weaker associations were found with water zones, and associations between a medium level of education and green and blue spaces were weaker (Figure 2A and Supporting Table S8A).

We found linear associations between household income and environmental exposures. Lower-income individuals were more likely to be exposed to all airborne pollutants (“<£18,000” vs “> £52,000”: β_NO_2__ = 2.13 (95% CI 2.06; 2.20); β_NO*_X_*_ = 4.90 (95% CI 4.74; 5.05); β_PM10_ = 0.34 (95% CI 0.32; 0.36); β_PM2.5_ = 0.35 (95% CI 0.34; 0.36)). Associations between intermediate household incomes and exposures to airborne pollutant were weaker suggesting a social gradient (“£18,000-£30,999” vs “>£52,000”: β_NO_2__ = 1.09 (95% CI 1.03; 1.15); β_NO*_X_*_ = 2.68 (95% CI 2.54; 2.83); β_PM10_ = 0.23 (95% CI 0.21; 0.25); β_PM2.5_ = 0.19 (95% CI 0.18; 0.20)). Lower-income individuals also experienced higher noise exposure (“<£18,000” vs “>£52,000” β_L,day_ = 0.42 (95% CI 0.37; 0.47); β_L,evening_ = 0.42 (95% CI 0.37; 0.47); β_L,night_ = 0.42 (95% CI 0.37; 0.47)). Individuals with a lower household income were less likely to reside in areas with higher percentage of gardens, green spaces or natural environment (“<£18,000” vs “>£52,000”: β_garden,1000m_ = −2.03 (95% CI −2.13; −1.93); β_greenspaces,1000m_ = −2.55 (95% CI −2.72; −2.38); β_natural,environment_ = −5.03 (95% CI −5.23; −4.82)) (Figure 2B and Supporting Table S8B).

Linear regression results showed that unemployed individuals had higher exposures to NO_2_ and NO*_X_* (Unemployed vs Employed: β_NO_2__ = 0.21 (95% CI 0.12;0.30); β_NO*_X_*_ = 0.32 (95% CI 0.11;0.53)) while retired individuals had lower exposures (Retired vs Employed: β_NO_2__ = −0.43 (95% CI −0.49; −0.37); β_NO*_X_*_ = −0.83 (95% CI −0.97; −0.69)). No association was found between the employment status and PM_10_ or PM_2.5_. Retirement was also associated with lower noise exposure (Retired vs Employed: β_L,day_ = −0.13 (95% CI −0.17; −0.09); β_L,evening_ = −0.13 (95% CI −0.17; −0.09); β_L,night_ = −0.13 (95% CI −0.17; −0.09)). No association was found between unemployment and noise exposure. Unemployed individuals were more likely to live in areas with less green space or natural environment (Unemployed vs Employed: β_natural,environment_ = −0.48 (95% CI −0.76; −0.21); β_greenspaces_ = −0.46 (95% CI −0.69; −0. 23)) while retired individuals were more likely to live in areas with more green space or natural environment (Retired vs Employed: β_natural,environment_ = 1.08 (95% CI 0.89;1.26); β_greenspaces_ = 0.78 (95% CI 0.62;0.94)) (Figure 2C and Supporting Table S8C).

#### Association between Respective SEP Indicators and Environmental Exposures in Rural Environments in England

Results of each SEP indicator and environmental exposures in rural residential localization in England (*N* = 50,178) are given in Figure 3 and Supporting Table S9.

**Figure 3:**
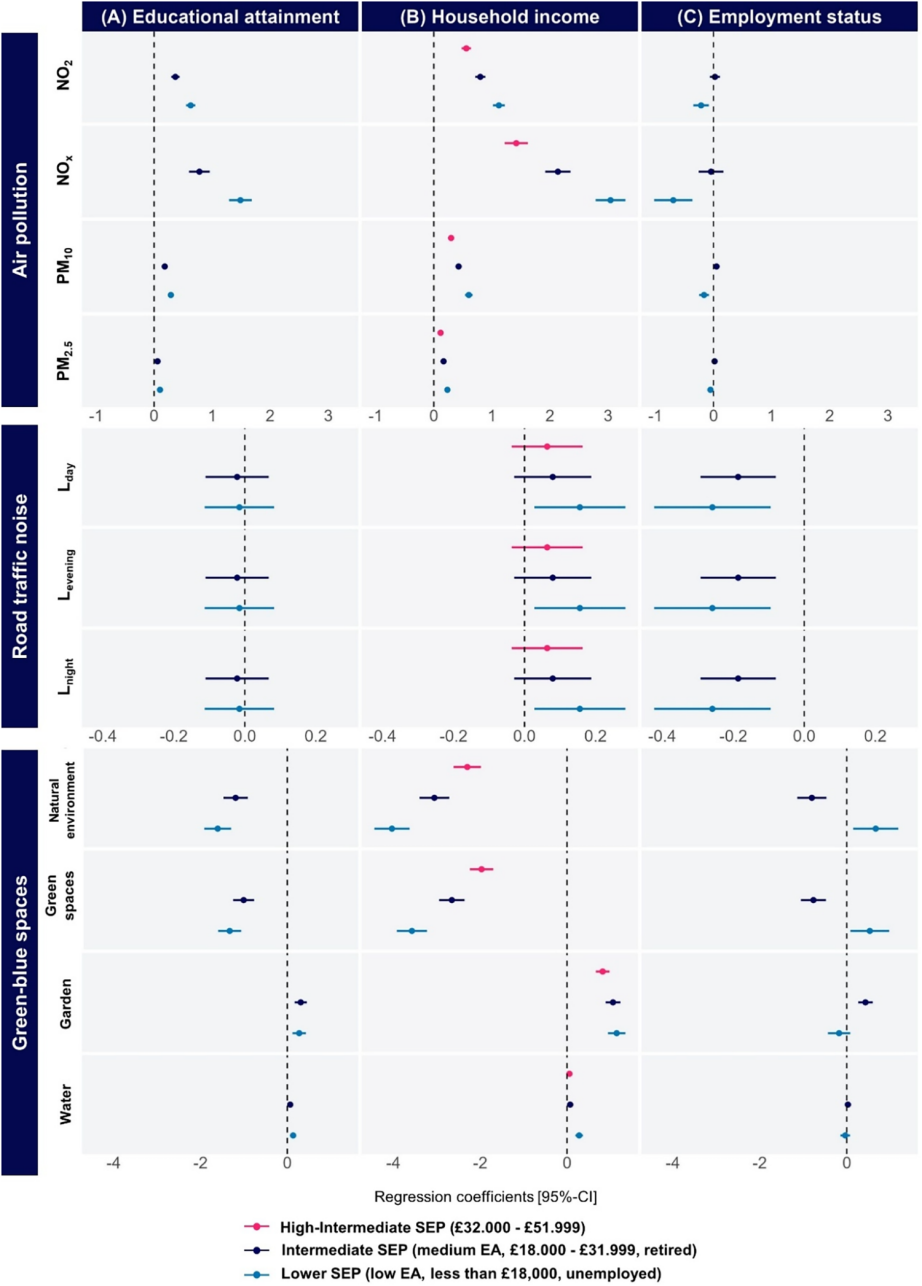
England rural areas: Forest plot of linear regression coefficients [95% confidence interval] for the association between (A) educational attainment, (B) household income, or (C) employment status and air pollution, road traffic noise, and green-blue spaces in rural areas for England (*N* = 50,178). β coefficients represent the difference in exposure to airborne pollutants, road traffic noise, and green-blue spaces compared to the reference group adjusted for covariates: for all environmental exposures, the most advantaged socioeconomic group was used as reference, and a positive association therefore indicates higher exposure in the less advantaged socioeconomic groups.

Overall, associations between educational attainment and environmental exposures in rural settings were broadly similar to urban ones but with several differences. For example, participants with either medium or low educational attainment were more exposed to airborne pollutants, compared to the urban areas, where only participants with lower educational attainment experienced higher exposure_._ Unlike in urban areas, individuals with a lower educational attainment were more likely to reside in areas with a higher percentage of gardens but less likely to access green spaces and the natural environment. Similar patterns were observed for individuals with medium educational attainment, although the effects were weaker compared to those with low educational attainment (Figure 3A and Supporting Table S9A).

A social gradient by household income persisted for airborne pollutants and proximity to the natural environment or green space, but associations between household income and noise exposures were weaker in rural settings. In contrast, the relationship between household income and proximity to garden was reversed in rural settings: lower-income households had greater proximity to garden (Figure 3B and Supporting Table S9B).

Overall, associations between employment status and environmental exposure in rural areas differed from those observed in urban settings. In rural areas, unemployment was associated with lower exposure to airborne pollutants. Specifically, unemployed individuals had lower levels of NO_2_, NO_*x*_, PM_10,_ and PM_2.5_, compared to employed individuals. Unemployed individuals also experienced lower levels of road traffic noise in rural areas. Additionally, they were more likely to reside near green or natural spaces, despite being further from gardens (Figure 3C and Supporting Table S9C).

#### Association between Household Income and Environmental Exposures After Sequential Adjustment for SEP

Results of household income and environmental exposures after sequential adjustment for SEP are given in Supporting Table S10. Sequential adjustment for educational attainment (Model 2A) and employment status (Model 2B) led to a modest attenuation of the associations observed in Model 1. When both variables were included simultaneously (Model 3), the results remained largely unchanged. These patterns were consistent in both urban and rural environments (Supporting Figures S2 and S3).

#### Association between Respective SEP Indicators and Environmental Exposures in Rural and Urban Environments in Wales and Scotland

The results of the analyses conducted in Wales (*N* = 16,617) and Scotland (*N* = 27,992) are shown in Supporting Tables S11 and S12, respectively, and Supporting Figures S4–S7.

In Wales, in urban areas, associations between SEP and environmental exposures provided similar patterns, though with weaker associations. Briefly, educational attainment and employment status had a mixed effect on airborne pollutants, while household income appears to have a stronger impact. Irrespective of the SEP indicator, no associations were observed with road traffic noise. Lower educational attainment and retirement were linked to a more natural environment, while low household income and unemployment were linked to a less natural environment (Supporting Table S11A and Supporting Figure S2). In rural areas, relationships between SEP and environmental exposure were broadly consistent with those observed in England. Greater exposure to airborne pollutants, road traffic noise, and reduced availability of natural environments were observed among individuals with lower educational attainment and those with a lower income. Mixed effects were found for employment status (Supporting Table S11B and Supporting Figure S3).

In Scotland, the social patterning of environmental exposures was more heterogeneous. In urban areas, we found that having a lower education and being retired were associated with being less exposed to NO_2_ and NO*_X_*, while unemployment and lower household income were associated with higher exposure to airborne pollutants. Lower educational attainment and retirement were associated with having a greater availability of the natural environment (Supporting Table S12A and Supporting Figure S4). Little to no associations were found between either educational attainment or employment status with noise population, while participants with lower household income experienced higher road traffic noise. In rural areas, results mirrored those in England, except for road traffic noise, where negative associations were observed between each SEP and road traffic noise (Supporting Table S12B and Supporting Figure S5).

## Discussion

Our findings show that many of the environmental exposures we examined are socially patterned in both urban and rural areas in the UK Biobank. Across all socioeconomic position markers (educational attainment, household income, and employment status) and regardless of the living area (urban or rural), individuals at disadvantaged SEP generally experienced higher exposure to airborne pollutants and limited availability of green and blue spaces. Household income was the only SEP marker associated with road traffic noise, with lower household income experiencing the worst noise pollution. The relationship between employment status and environmental exposures varied between urban and rural areas. In rural areas, unemployed individuals experienced better air quality in terms of residential air pollutants and had less availability of green spaces or a natural environment, while in urban areas, this pattern did not hold. These results highlight the complexity of employment status, which may be influenced by how employment categories were defined and is strongly related to the participant’s age. Our results suggest that household income is a key determinant of environmental inequalities more strongly than education or employment status in both urban and rural areas. This was further confirmed by subsequent analysis in which SEP variables were added to the model in chronological order. We observed that associations between environmental exposures and household income were independent of education and employment status (Supporting Figures S6 and S7 and Supporting Table S10). Overall, the consistent relationship between disadvantaged SEP and poorer environmental exposures suggests that the SEP is a key determinant of the external exposome.

Despite some variation, our results were largely consistent across England, Scotland, and Wales, reinforcing the robustness of these findings. However, the observed geographical [urban/rural] and national [England/Wales/Scotland] differences also underscore how socioeconomic factors, such as educational attainment, household income, and employment status, are linked to environmental exposures in distinct ways across geographical contexts.

Our findings are consistent with previous research on social inequalities from environmental exposures. Numerous studies have explored the relationships between SEP and air pollution. A recent review reported that for most of the pollutants (including PM_2.5_, PM10, NO_2_, and NO*_X_*), deprived areas experience the worst air quality across Europe.^40^ In the UK, socioeconomic inequalities in exposure to air pollution have been well documented. Milojevic et al. reported that average PM concentrations are consistently higher in the most socially deprived areas, regardless of whether they are in rural or urban neighborhoods.^41^ By contrast, a nationwide study in England found weak, inconsistent links between social deprivation and air pollution.^42^ A study in London in 2011 using the Whitehall II cohort study examined SEP at both the individual and geographical levels, and they found higher exposure to air pollution among individuals with lower SEP and more deprived areas.^43^ However, they also highlighted inconsistencies in the role of education, areas with higher educational levels had the highest exposure, and educational attainment at the individual level was the only SEP markers with no clear association with air pollution.^43^ Moreover, most of these studies assess SEP at a geographical level. Our study provides new evidence by using measures of SEP at the individual level and by specifically examining how individual SEP influences spatially determined environmental exposure such as air pollution.

Two studies investigating the urban exposome among pregnant women in various European cities found heterogeneous associations between SEP and urban environmental exposures. Robinson et al. reported that lower SEP was associated with higher levels of air pollutants in Bradford, Nancy, and Valencia, but with lower exposure levels in Oslo, Poitiers, and Sabadell.^26^ The study also underlined inconsistent patterns for noise pollution and access to green spaces across Europe. Similarly, Pizzi et al. found strong associations across urban environment factors and indicators of maternal education and household income, but these associations varied by environmental domain and cities.^27^ For example, in Oslo and Valencia, lower incomes were associated with poorer air quality, a less healthy food environment, greater density, and limited access to natural spaces, whereas inverse associations were observed in Turin and Sabadell.

Some studies have found higher air pollution levels in more socially disadvantaged populations in cities like Malmö (children only), Oslo,^44^ Strasbourg,^45^ Netherlands,^46^ and London.^47^ In contrast, findings from Rome^48^ suggest that exposure to traffic emissions was higher among those with higher area-based income and SEP. These studies rely on area-level deprivation indices or neighborhood-level socioeconomic indicators to assess the social patterning of pollution exposure. Additionally, studies from Italy^49^ and Spain during pregnancy,^50^ using individual SEP indicators such as education or income, reported higher pollution exposure among more affluent groups. In our study, we found that, in both urban and rural areas, lower educational attainment and household income were associated with higher exposure to NO_2_, NO*_X_*, PM_10_, and PM_2.5_. In urban areas, unemployed individuals had higher exposure to NO_2_ and NO*_X_* only, while retired individuals had lower exposure to both. In contrast, in rural areas, unemployed individuals had lower exposure to NO_2_ and NO*_X_*. We also observed differences between countries. Overall, our study suggests that, in the UK, social inequalities in exposure to airborne pollutants may not always be significant or consistently negative, highlighting the complexity and the multidimensional nature of the individual-environment interconnection.

There is limited evidence of the social patterning of road traffic noise. A study in Greater London found that more deprived neighborhoods are exposed to higher levels of road traffic noise.^51^ Another study in England identified significant associations between lower educational attainment, living more deprived areas and job-related factors with higher level of noise complaints.^52^ However, mixed results were observed in four urban areas in England concerning the social patterning of transportation road traffic noise.^53^ Additionally, a WHO systematic review focusing on road traffic noise concluded that while results are mixed, they still suggest social inequalities in exposures to noise.^19^ In our study, we observed different patterns of noise exposure in urban and rural areas. In urban areas, educational attainment and employment status were not associated with noise exposure. However, lower household income showed a strong and consistent link to higher noise exposure; retired individuals were more likely to experience lower noise levels compared to employed people. In rural areas, no significant association was found between educational attainment and road traffic noise. In contrast, lower income individuals, particularly those in the lowest income group, were still more likely to be exposed to higher noise levels. Unemployed and retired individuals had lower noise exposure compared to the employed individuals. Overall, these findings highlight the complex interplay among socioeconomic position, geographic location, and exposures to noise, with different patterns emerging depending on urban versus rural contexts.

Deprived areas often have higher residential density, with land use being dominated by retail or industrial zones, limiting the availability of green spaces and recreation centers.^54^ A systematic review by Schüle et al. highlights a consistent pattern of social inequalities in the availability of green and blue spaces within the WHO European Region, particularly at the area level.^20^ Studies in Germany^55^ and Southern Europe^56^ found that neighborhoods with disadvantaged SEP had less availability of green space and faced greater barriers to quality and accessibility. In our study, we found that in urban areas, individuals with lower educational attainment tended to have fewer private gardens but greater availability of green spaces and natural environments. Lower household income was more strongly associated with reduced availability of gardens, green spaces, and nature compared to education. Employment status also played a role, with unemployed individuals having less availability of green space, while retired individuals experienced greater availability compared with employed individuals. In rural areas, we observed that individuals with a lower educational attainment and income had greater availability of private gardens but reduced availability of green space and natural environments compared with the higher socioeconomic position group, showing a clear social gradient. Unemployed individuals were less likely to live near gardens but more likely to reside in areas with green space or natural environments. These results contrast how different socioeconomic factors, educational attainment, household income, and employment status, are linked to green space through distinct pathways in both rural and urban areas.

### Strengths

The main strength of our study lies in its comprehensive investigation of a wide range of individual-level socioeconomic position indicators and environmental exposures spanning three distinct regions within a large sample size of >380,000 participants from the UK Biobank. This allowed us to conduct stratified analyses based on urbanicity within each country, providing the opportunity to examine how the interplay between social factors and environmental exposures varies across countries and across urban and rural contexts. Using three distinct SEP markers allowed us to account for different social dimensions and different life stages. As defined by Galobardes et al.,^57^ educational attainment marks the transition from early life, shaped by the parents’ SEP, to young adulthood’s SEP, while being a strong determinant of later income and occupation. Education can reflect an individual’s material, intellectual, and cultural resources. Income, representing middle-to-late SEP, provides a direct measure of material resources. Better material conditions can translate, through mediating pathways, to a better health status. Finally, Occupation, which also provides a measure of middle-to-late SEP, provides insight into both economic standing and psychosocial work conditions. Taken together, these complementary markers offer a more comprehensive understanding of the social position and the mechanisms in play regarding environmental inequalities.

### Limitations

Despite the strengths of our study, several limitations should be considered. First, as with any cohort study, selection bias is inherent. The UK Biobank is known to have an overrepresentation of individuals with higher educational attainment and healthier lifestyle.^58^ Our additional selection criteria may have further accentuated this pattern, which likely underestimates the true magnitude of socioeconomic inequalities in environmental exposures. Moreover, the uneven geographical distribution of assessment centers, in Scotland (3 centers) and Wales (2 centers) and in the Southwest or the Northwest of England, results in an under-representation of these populations, which may limit the generalizability of our findings to the UK as a whole. Second, it is likely that other relevant pathways contribute to these associations and remain unaccounted for. Together with possible residual confounding due to unmeasured confounders, this may have led to over- or underestimation of the observed associations. Given the cross-sectional nature of the data, we cannot be certain of the causation. Furthermore, we employed a conceptual model for all three environmental exposure domains that may have oversimplified the complex mechanisms at play, which could explain the strength of some observed associations. Third, the categories of employment status in our study are quite heterogeneous. The ’employed’ category in particular encompasses a wide range of occupations and work conditions. Moreover, the inclusion of volunteers and students in the unemployed category may dilute the effects of true unemployment. The retired category may also include early retirees, who could have either a high SEP or poor health, and retirement is strongly related to age, further complicating the interpretation of the results. While this approach allowed us to include both unemployed and retired individuals, it may have oversimplified the employment status variable, potentially masking important differences within the employed group. As a result, findings related to this indicator should be interpreted with caution.

Precaution is warranted when interpreting our findings within a life-course framework. Although we used three indicators of SEP to represent different life stages, all were measured at the baseline. Employment status and household income primarily reflect current socioeconomic conditions, rather than clearly distinct phases of middle-to-late adulthood, especially given the age variation among participants at enrollment. These measures offer insight into different social dimensions, but they do not fully reflect life course SEP. Ideally, longitudinal data or additional indicators, such as parental SEP, would allow for a more robust assessment of life course SEP influences on environmental exposures.

Additionally, the environmental exposure variables used were several years old, warranting caution when extrapolating the results to the present day. Levels of air pollution decreased greatly between 2006 and 2025.^59^ Although past exposures may still have adverse health effects, the associations we observed could be weaker or not fully representative of the current conditions. We lacked data on participants’ spatial mobility during the baseline period (2006–2010), preventing us from accounting for changes in exposure due to relocation. Although GLUD 2005 provided the only national coverage of detailed small-area green space data, it was based on a limited number of sources. Lastly, road traffic noise levels estimated from outdoor residential addresses, not at the individual level, introduced uncertainty due to factors such as time spent away from home, building structure, and window usage, which may have led to a potential underestimation of our observed associations. Finally, relying only on residence-based exposure may lead to biased results, as individuals spend significant time away from home in places like schools, workplaces, and public spaces.^60^ Future work incorporating longitudinal designs and finer occupational classifications, and considering mediators, could help address these issues more comprehensively.

### Perspectives

Our study aligns with the growing field of social exposome research. There is a need to further explore other social factors across the life-course and environmental exposures to better understand the complex relations at play. Future research should further investigate the specific roles of different SEP markers in shaping environmental exposure inequalities. Additionally, examining the interaction between individual SEP and the broader environment could provide insights into potential protective or detrimental effects on health.

In conclusion, our study provides consistent evidence of social inequality in environmental exposures. Both in urban and rural contexts, individuals at disadvantaged socioeconomic positions experience overall poorer air quality, higher road traffic noise, and reduced availability of green spaces. Moreover, our findings highlight how different socioeconomic factors measured at the individual level, such as educational attainment, household income, and employment status, are linked to environmental exposures in distinct ways in England, Scotland, and Wales and in both urban and rural areas.

## Supplementary Material





## Notes

Overall study workflow and data availability across countries (Supporting Figure S1); forest plots of linear regression coefficients for associations between household income and air pollution, road traffic noise, and green–blue spaces in urban and rural areas of England, respectively, under progressively adjusted models (Supporting Figures S2 and S3); associations between each socioeconomic indicators and each environmental exposures for urban and rural areas of Wales (Supporting Figures S4 and S5); corresponding results for urban and rural areas of Scotland (Supporting Figures S6 and S7) (PDF)
Socioeconomic indicators, environmental exposures, and covariates included in the analysis (Supporting Tables S1–S3); characteristics of the analytical sample compared with the full UK Biobank cohort by country (Supporting Table 5); bivariate associations between confounders, socioeconomic position indicators, social determinants, and environmental exposures, stratified by urban and rural areas in England (Supporting Tables S6A–B and S7); linear regression results for associations between socioeconomic indicators and environmental exposures in urban and rural areas of England (Supporting Tables S8–S10); extend these analyses to Wales and Scotland, respectively (Supporting Tables S11 and S12) (XLSX)

## Abbreviations

SEP: socioeconomic position
