# Supplementary material for: Education, Employment, Income, and Urban–Rural Differences as Drivers of Social Inequalities in Environmental Exposures: Evidence from the UK Biobank
Source: Environ Sci Technol. 2026 Jan 22;60(4):3326–38. doi: 10.1021/acs.est.5c08849 (PMC12874515; doi:10.1021/acs.est.5c08849)
Supplement: Supplementary file 1 [file es5c08849_si_001.pdf]

Education, employment, income and urban-rural differences as drivers of social inequalities in environmental exposures: evidence from the UK Biobank

Gauthier Pereira[1], Benoît Lepage[1], Kees de Hoogh[2,3], Ruben Colindres Zuehlke[4], Fernando Guntoro[4], Lola Neufcourt[1], David Tang[4], Rin Wada[4], Roel Vermeulen[5], Michelle Kelly-Irving[1], Cyrille Delpierre[1], Marc Chadeau-Hyam[4], Raphaële Castagné[1]*

1. EQUITY Team, Centre d'Epidémiologie et de Recherche en santé des POPulations CERPOP-UMR1295, Inserm–Université de Toulouse, 31400 Toulouse, France.
2. Swiss Tropical and Public Health Institute, 4123 Allschwil, Switzerland.
3. University of Basel, 4001 Basel, Switzerland.
4. Department of Epidemiology and Biostatistics, School of Public Health, Imperial College London, W12 0BZ London, UK
5. Institute for Risk Assessment Sciences, Utrecht University, 3584 CM Utrecht, the Netherlands.

Supplementary Figures

Supplementary Figure 1: Overview of the study workflow. PM_{10} and $PM_{2.5}$ were not available in Scotland as those airborne pollutants were modelized in a 400Km radius from London. Greenspaces, garden and water were not available for Scotland and Wales.

Supplementary Figure 2: Forest plot of linear regression coefficients [95% confidence interval] for the association between household income and (A) air pollution, (B) road traffic noise, and (C) green-blue spaces in urban areas for England (N=292,246). Lighter coloured triangles represent Model 1 adjusted for covariates (age, sex, country of birth and centre), solid circle represents Model 2A further adjusted for educational attainment (Model 1 + educational attainment), darker coloured circles represent Model 2B further adjusted for employment status (Model 1 + Employment status), solid triangle represents Model 3 further adjusted for educational attainment and employment status (Model 1 + educational attainment + employment status). The 'More than £52,000' group was used as reference, pink represent the '£32,000-£51,999', dark blue represents the '£18,000-£31,999'group and light blue the 'less than £18,000'. The employed group was used as reference, dark blue represents the 'retired' group and light blue 'Unemployed' group.

Supplementary Figure 3: Forest plot of linear regression coefficients [95% confidence interval] for the association between household income and (A) air pollution, (B) road traffic noise, and (C) green-blue spaces in rural areas for England (N=50,178). Lighter coloured triangles represent Model 1 adjusted for covariates (age, sex, country of birth and centre), solid circle represents Model 2A further adjusted for educational attainment (Model 1 + educational attainment), darker coloured circles represent Model 2B further adjusted for employment status (Model 1 + Employment status), solid triangle represents Model 3 further adjusted for educational attainment and employment status (Model 1 + educational attainment + employment status). The 'More than £52,000' group was used as reference, pink represent the '£32,000-£51,999', dark blue represents the '£18,000-£31,999'group and light blue the 'less than £18,000'. The employed group was used as reference, dark blue represents the 'retired' group and light blue 'Unemployed' group.

Supplementary Figure 4: Forest plot of linear regression coefficients [95% confidence interval] for the association between educational attainment, household income or employment status and (A) air pollution, (B) road traffic noise, and (C) green-blue spaces in urban areas for Wales (N=14,089).

Supplementary Figure 5: Forest plot of linear regression coefficients [95% confidence interval] for the association between educational attainment, household income or employment status and (A) air pollution, (B) road traffic noise, and (C) green-blue spaces in rural areas for Wales (N=2,528).

Supplementary Figure6: Forest plot of linear regression coefficients [95% confidence interval] for the association between educational attainment, household income or employment status and (A) air pollution, (B) road traffic noise, and (C) green-blue spaces in urban areas for Scotland (N=25,591).

Supplementary Figure 7: Forest plot of linear regression coefficients [95% confidence interval] for the association between educational attainment, household income or employment status and (A) air pollution, (B) road traffic noise, and (C) green-blue spaces in rural areas for Scotland (N=2,401).

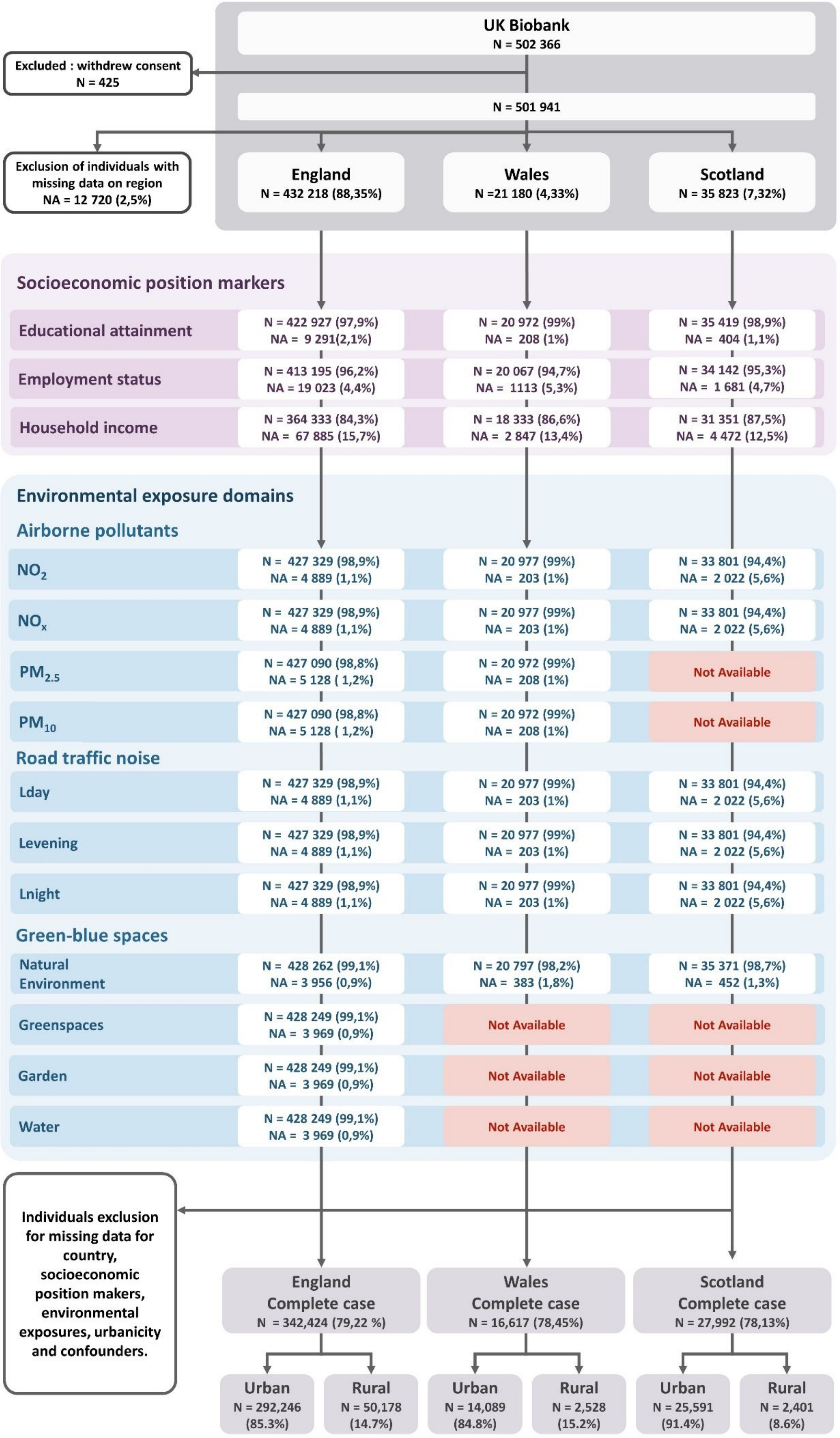

Supplementary Figure 1: Overview of the study workflow. PM_{10} and $PM_{2.5}$ were not available in Scotland as those airborne pollutants were modelized in a 400Km radius from London. Greenspaces, garden and water were not available for Scotland and Wales.

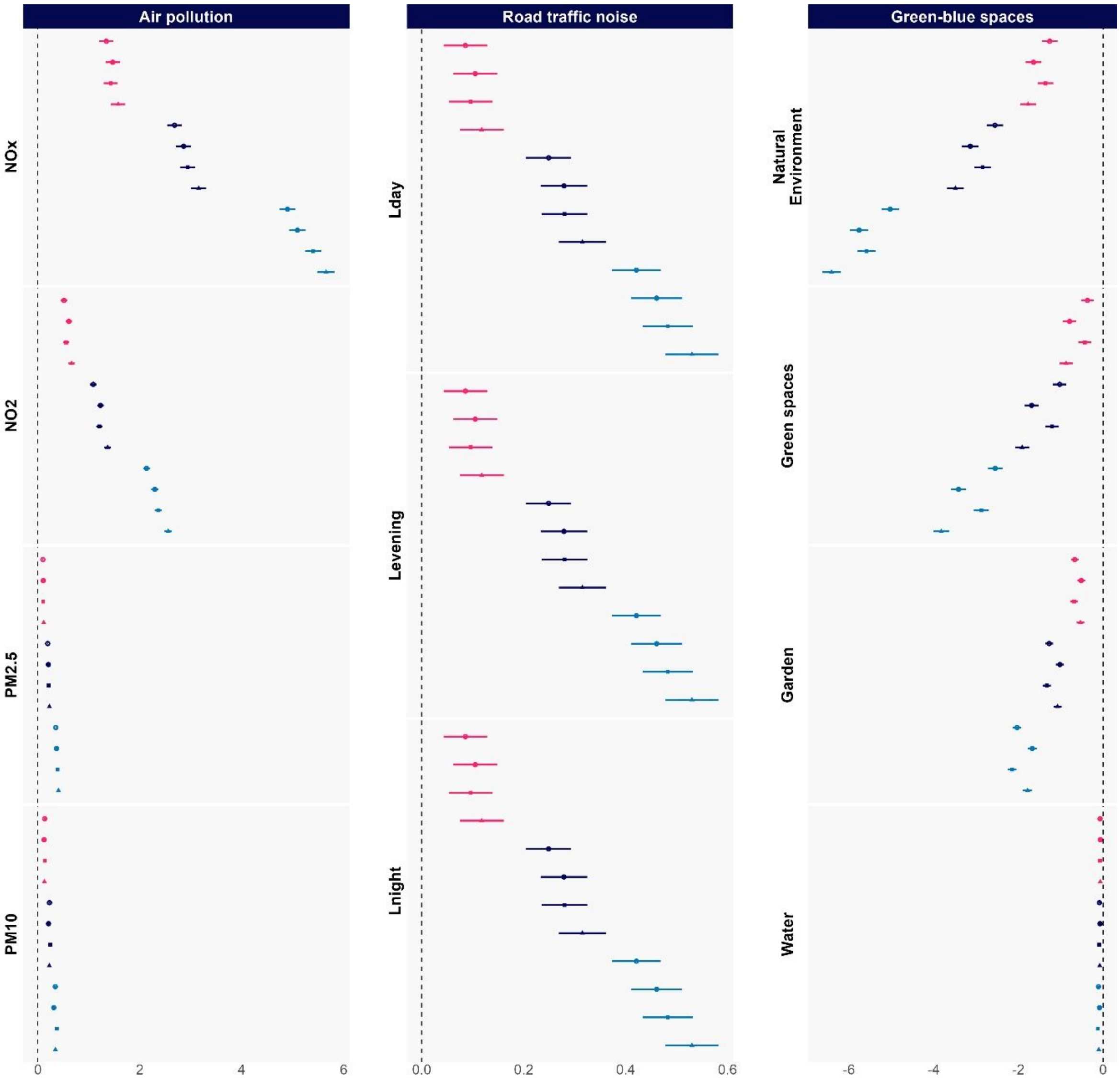

Supplementary Figure 2: Forest plot of linear regression coefficients [95% confidence interval] for the association between household income and (A) air pollution, (B) road traffic noise, and (C) green-blue spaces in urban areas for England (N=292,246). Unfilled circles represent Model 1 adjusted for covariates (age, sex, country of birth and centre), solid circle represents Model 2A further adjusted for educational attainment (Model 1 + educational attainment), solid squares represent Model 2B further adjusted for employment status (Model 1 + Employment status), solid triangle represents Model 3 further adjusted for educational attainment and employment status (Model 1 + educational attainment + employment status). The 'More than £52,000' group was used as reference, pink represent the '£32,000-£51,999', dark blue represents the '£18,000-£31,999'group and light blue the 'less than £18,000'. The employed group was used as reference, dark blue represents the 'retired' group and light blue 'Unemployed' group.

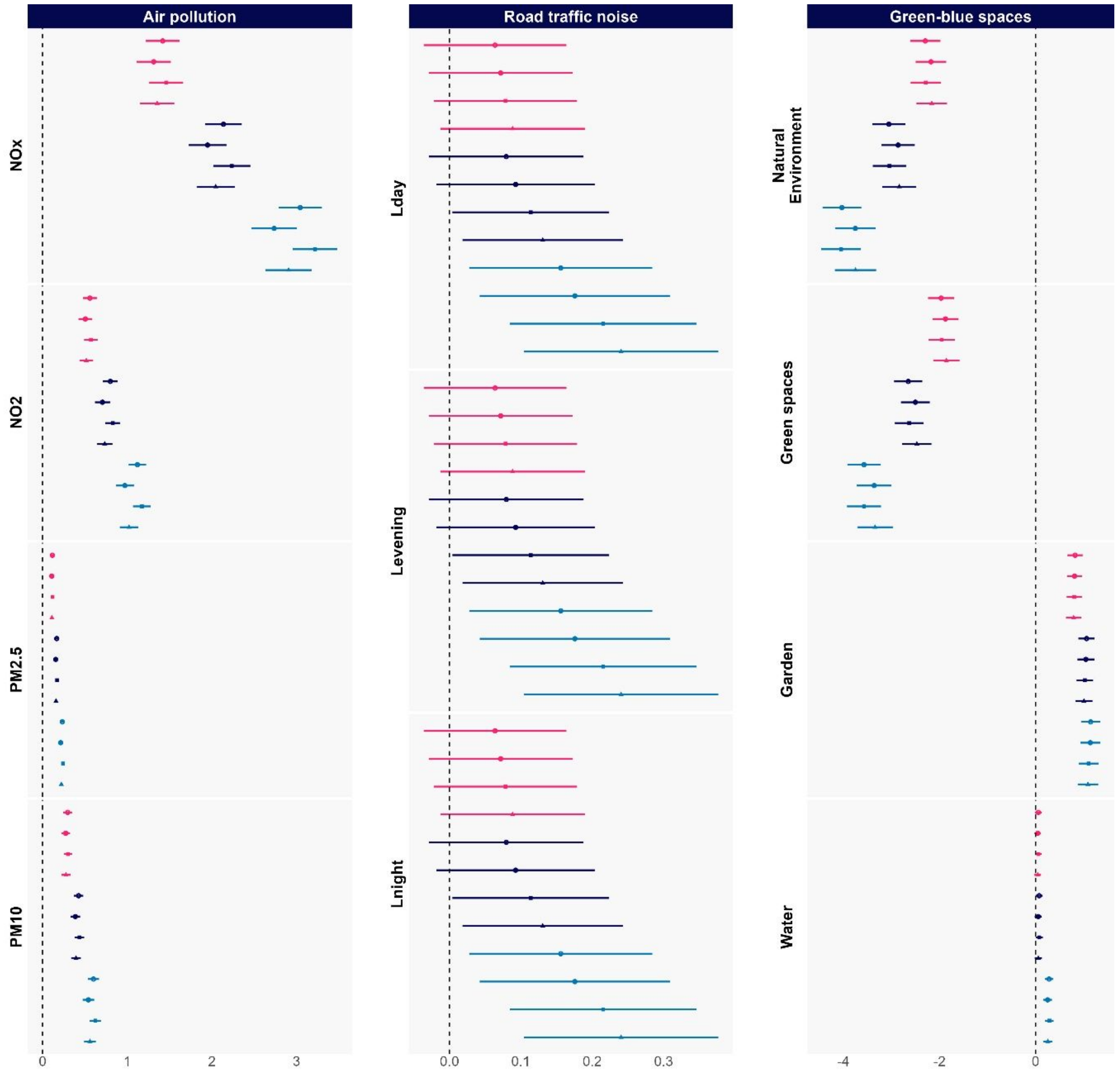

Supplementary Figure 3: Forest plot of linear regression coefficients [95% confidence interval] for the association between household income and (A) air pollution, (B) road traffic noise, and (C) green-blue spaces in rural areas for England (N=50,178). Unfilled circles represent Model 1 adjusted for covariates (age, sex, country of birth and centre), solid circle represents Model 2A further adjusted for educational attainment (Model 1 + educational attainment), solid squares represent Model 2B further adjusted for employment status (Model 1 + Employment status), solid triangle represents Model 3 further adjusted for educational attainment and employment status (Model 1 + educational attainment + employment status). The 'More than £52,000' group was used as reference, pink represent the '£32,000-£51,999', dark blue represents the '£18,000-£31,999' group and light blue the 'less than £18,000'. The employed group was used as reference, dark blue represents the 'retired' group and light blue 'Unemployed' group.

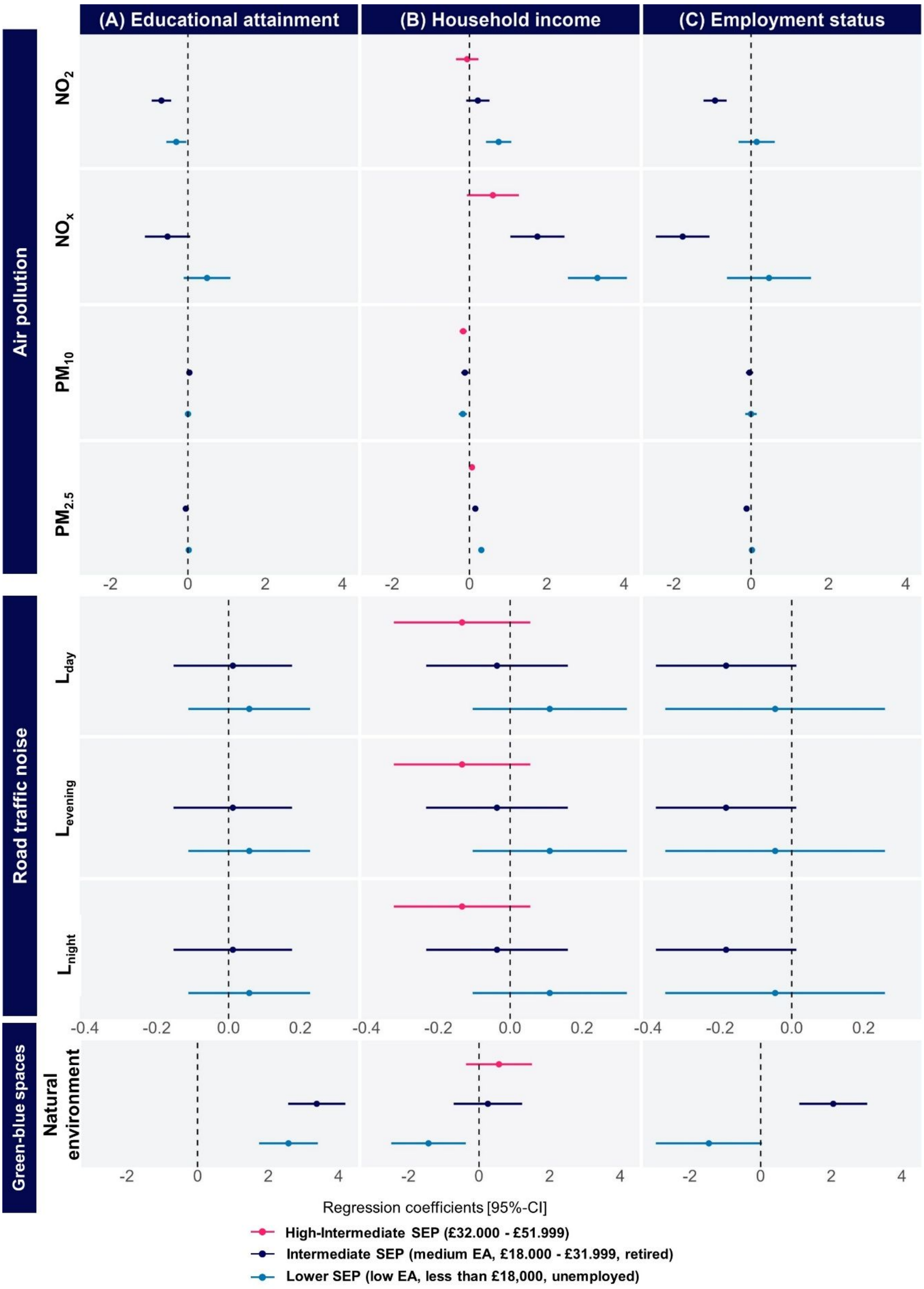

Supplementary Figure 4: Forest plot of linear regression coefficients [95% confidence interval] for the association between educational attainment, household income or employment status and (A) air pollution, (B) road traffic noise, and (C) green-blue spaces in urban areas for Wales (N=14,089).

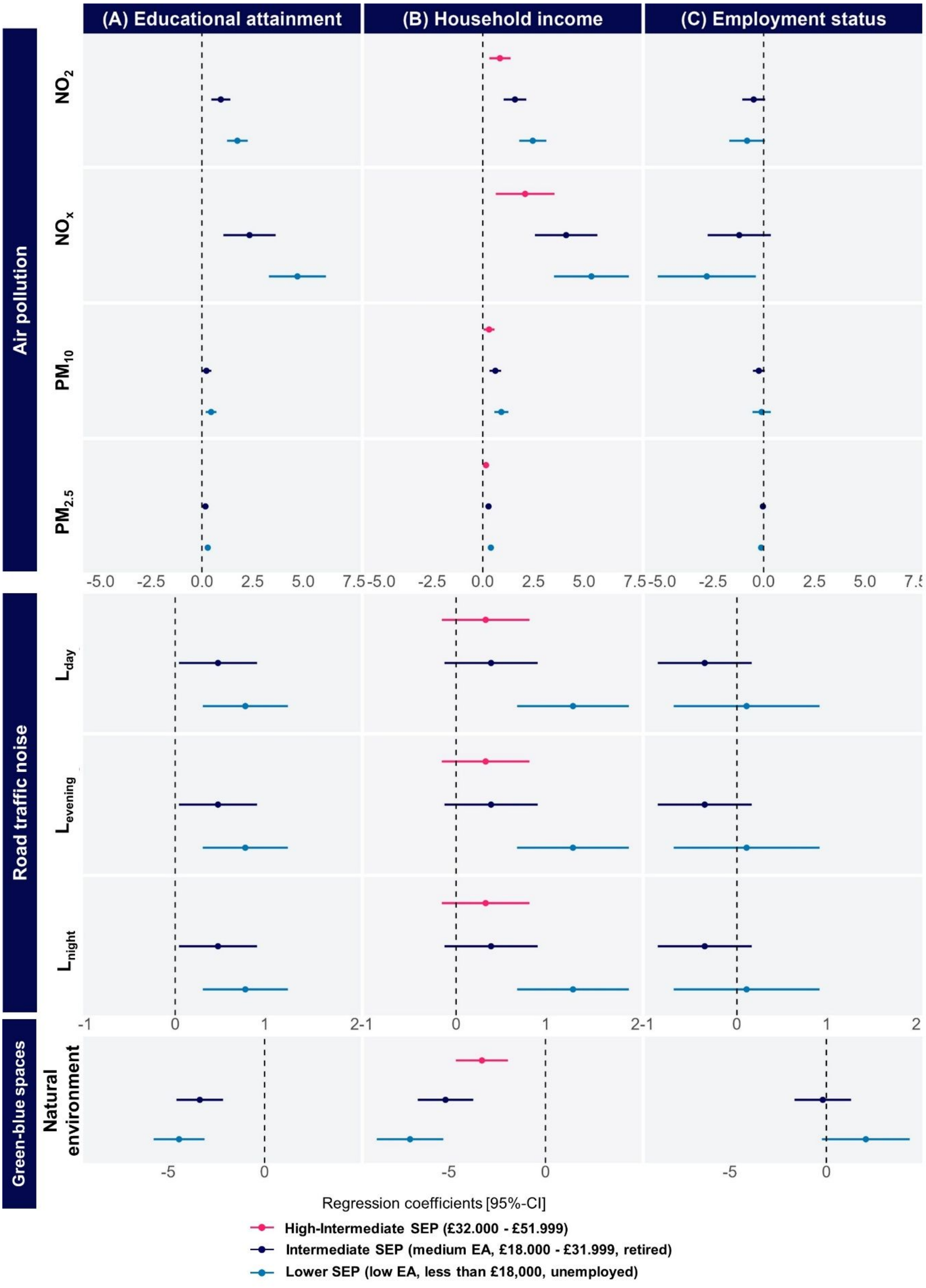

Supplementary Figure 5: Forest plot of linear regression coefficients [95% confidence interval] for the association between educational attainment, household income or employment status and (A) air pollution, (B) road traffic noise, and (C) green-blue spaces in rural areas for Wales (N= 2,528).

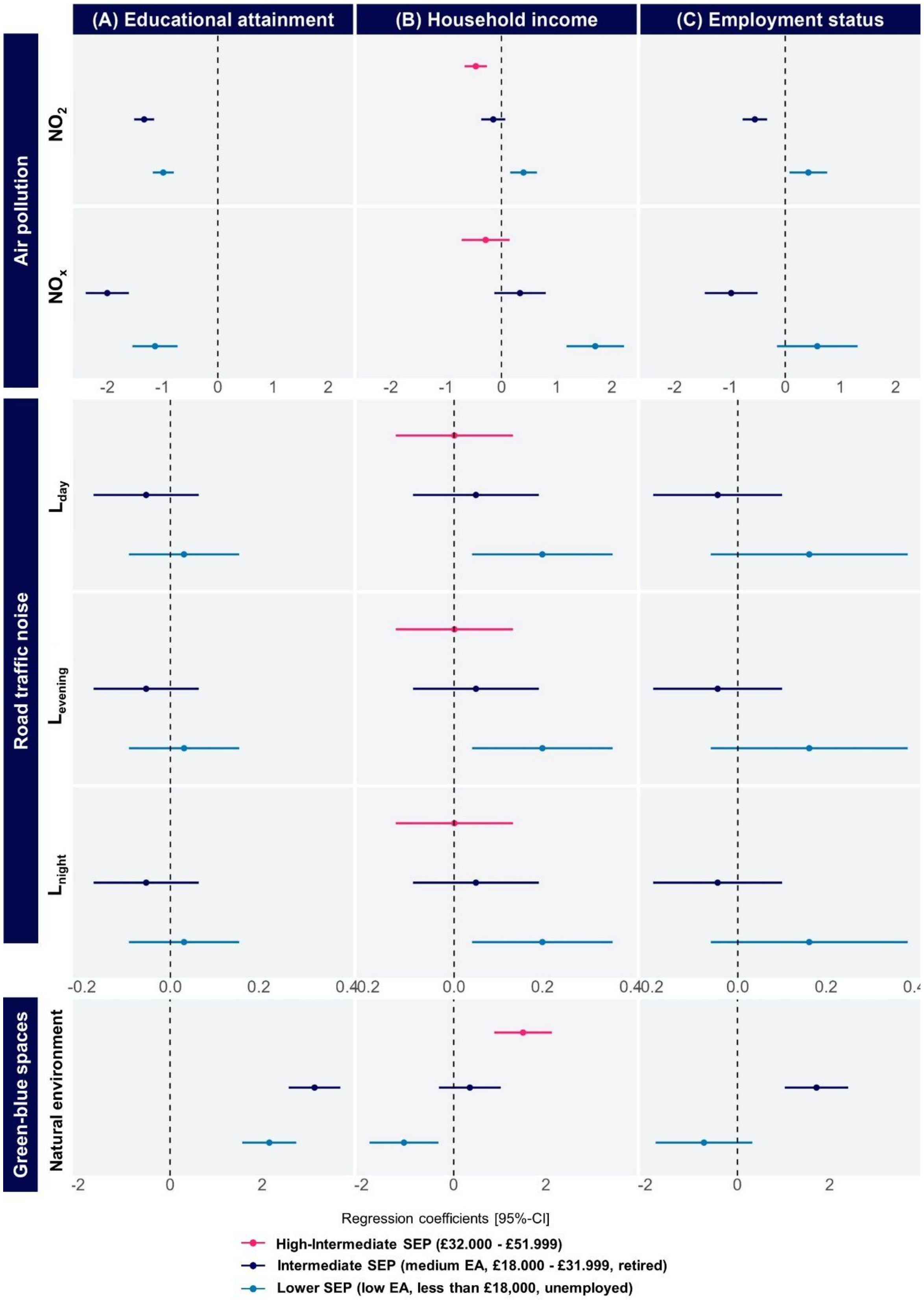

Supplementary Figure 6: Forest plot of linear regression coefficients [95% confidence interval] for the association between educational attainment, household income or employment status and (A) air pollution, (B) road traffic noise, and (C) green-blue spaces in urban areas for Scotland (N= 25,591).

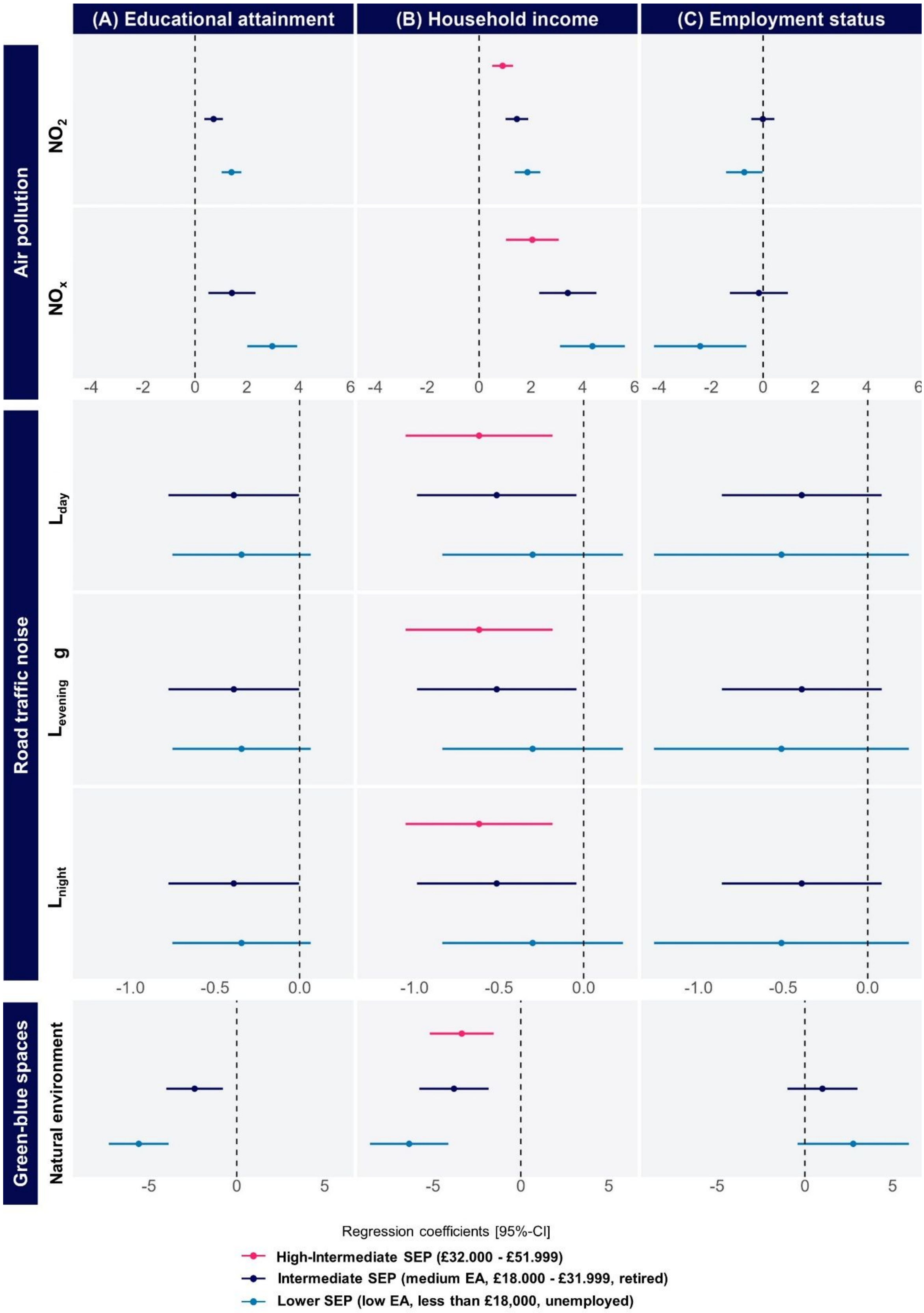

Supplementary Figure 7: Forest plot of linear regression coefficients [95% confidence interval] for the association between educational attainment, household income or employment status and (A) air pollution, (B) road traffic noise, and (C) green-blue spaces in rural areas for Scotland (N= 2,401).
